# Supplementary material for: The genomic impact of historical hybridization with massive mitochondrial DNA introgression
Source: Genome Biol. 2018 Jul 30;19:91. doi: 10.1186/s13059-018-1471-8 (PMC6065068; doi:10.1186/s13059-018-1471-8)
Supplement: Supplementary file 2 — Figure S1. Distribution of differential levels of average introgression between the five northern and five southern individuals across the 1000 simulations of mitochondrial introgression. Figure S2. Power and false discovery rate of the relative node depth method for inferring introgression. Figure S3. Empirical distribution of nuclear introgression frequencies inferred with RND. Figure S4. Introgression frequency distribution of mitonuc and background genes. Figure S5. Variation of the proportion of introgression across individuals for autosomes and the X chromosome. Figure S6. Correlation between prevalence of introgression (estimated with the ELAI method) and relative distance to the centromere for all chromosomes. Figure S7. Correlation between prevalence of introgression (estimated with the ELAI method) and relative distance to a the centromere and b the chromosome center for each chromosome category. Figure S8. Correlation between introgression tract size and geography. Figure S9. Expected introgression frequency distribution in a sample of ten L. granatensis individuals with the same geographic origin as the ten samples used in this study, considering empirical mtDNA introgression frequencies. (PDF 1177 kb) [file 13059_2018_1471_MOESM2_ESM.pdf]

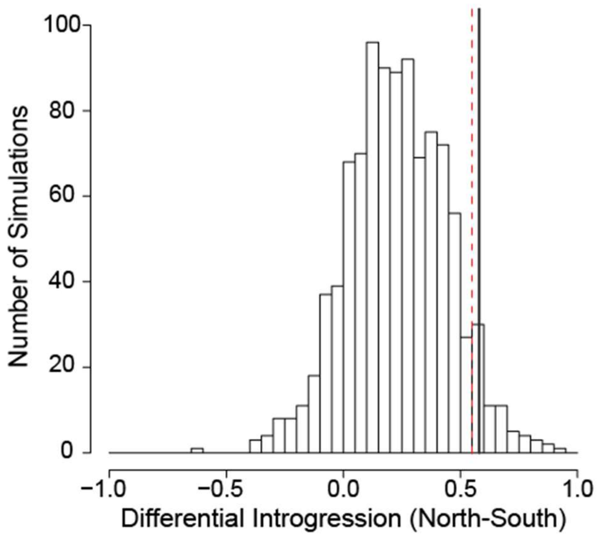

Figure S1. Distribution of differential levels of average introgression between the 5 northern and the 5 southern individuals across the 1000 simulations of mitochondrial introgression. The vertical red dashed line indicates the empirical difference while the solid black line represents the 95% percentile value of the simulated distribution.

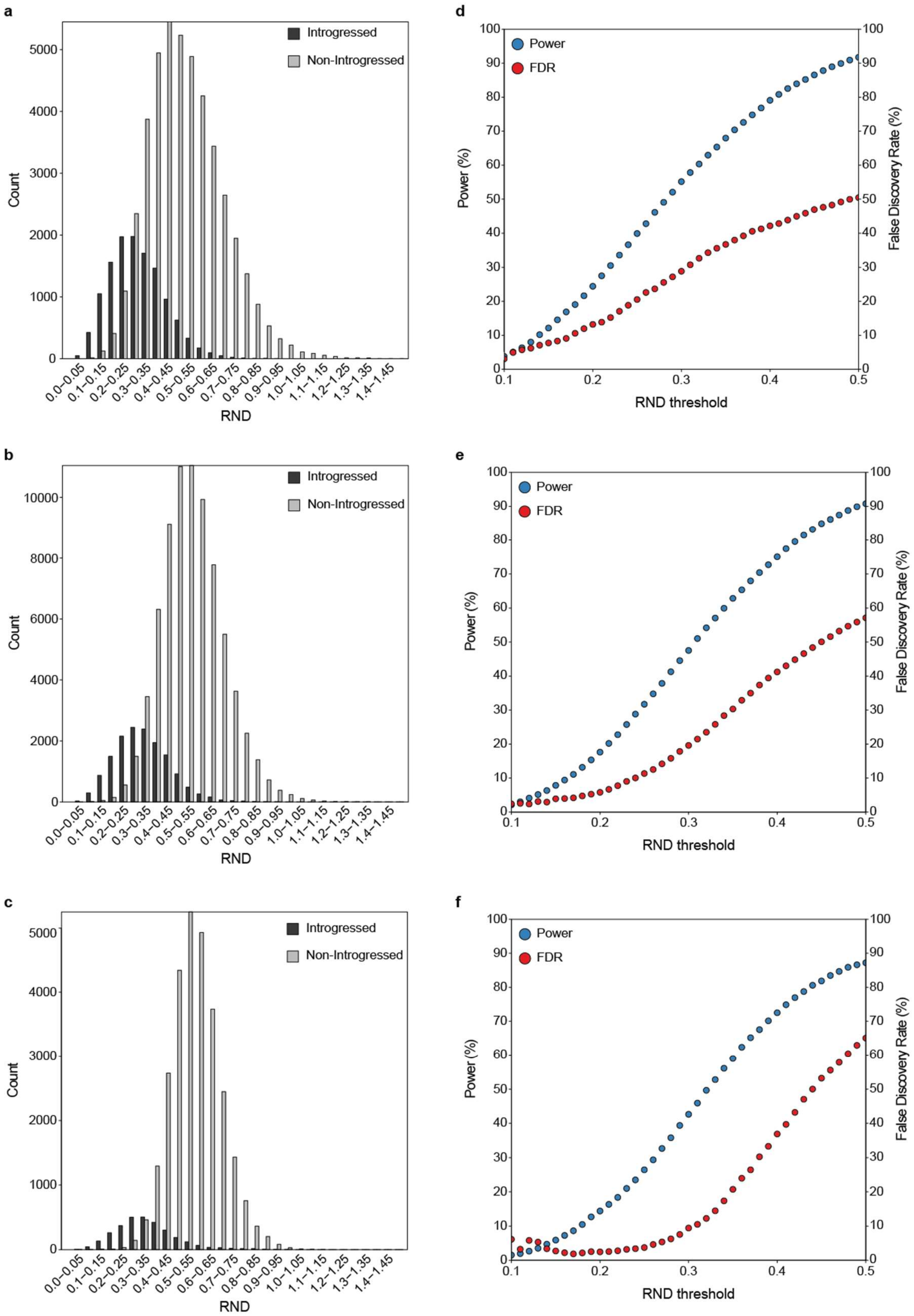
a
Introgressed
Non-Introgressed
Count
RND
b
Introgressed
Non-Introgressed
Count
RND
c
Introgressed
Non-Introgressed
Count
RND
d
Power
FDR
Power (%)
False Discovery Rate (%)
RND threshold
e
Power
FDR
Power (%)
False Discovery Rate (%)
RND threshold
f
Power
FDR
Power (%)
False Discovery Rate (%)
RND threshold

Figure S2. Power and False Discovery Rate (FDR) of the Relative Node Depth (RND) method for inferring introgression detected with the ELAI method. (a-c) Distribution of RND minimum values across all individuals for windows completely within ELAI introgression fragments (black) and windows not overlapping such fragments (grey), for RND window sizes of (a) 10kb, (b) 20kb and (c) 50kb. (d-f) Estimates of Power (blue) and FDR (red) as a function of RND minimum thresholds used to define RND windows as introgressed, for the three RND window sizes.

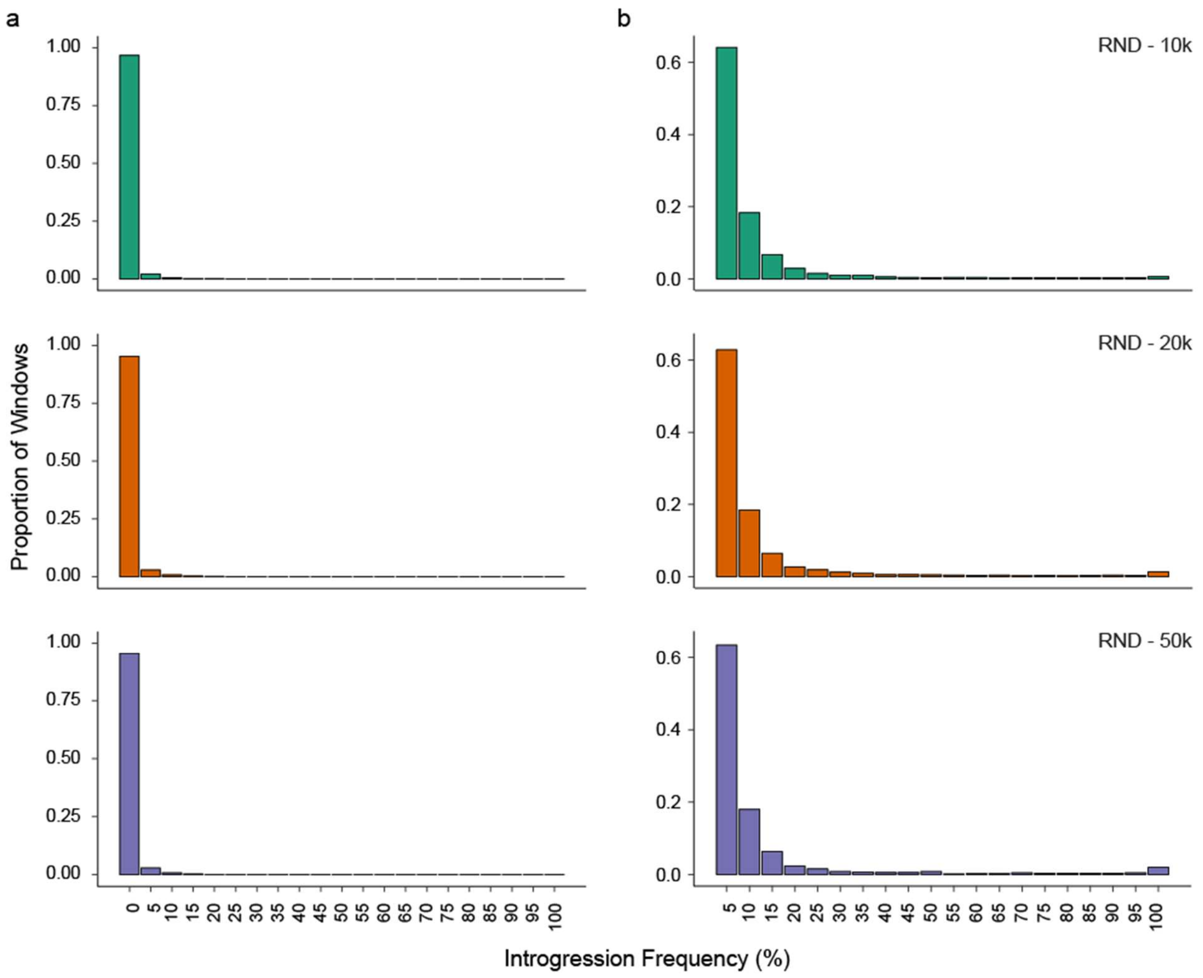

Figure S3. Empirical distribution of nuclear introgression frequencies inferred with RND with different window sizes: 10kb (green), 20kb (orange) and 50kb (blue). The frequency of windows (y-axis) at each introgression frequency (x-axis) was estimated either considering: (a) all possible introgression frequencies (that is, no introgression – 0% - to complete introgression – 100%) or (b) only considering windows with at least one haplotype introgressed (introgression frequency >= 5%). The latter representation reveals the tails of the distributions with some windows showing very high introgression frequencies.

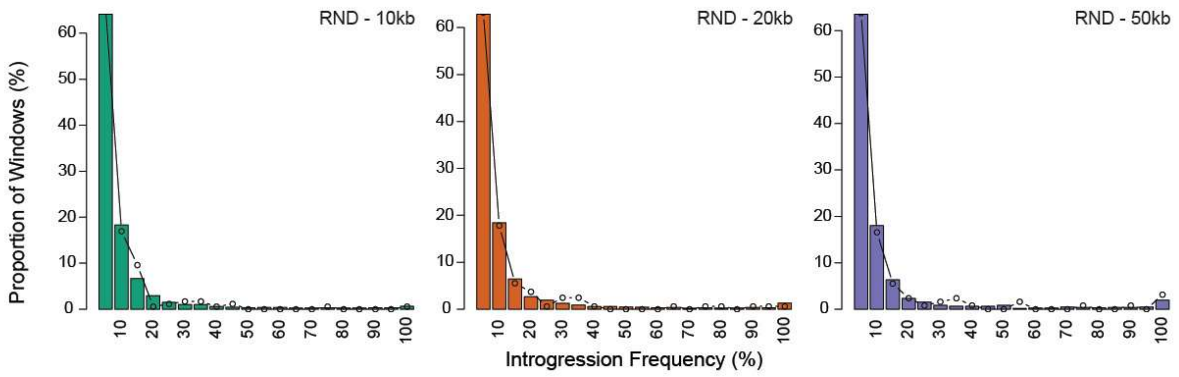

Figure S4. Introgression Frequency Distribution of “mitonuc” (lines) and background (bars) genes. Inferences using the RND method with three different window sizes are shown. The frequency distribution only considers windows with at least one haplotype introgressed (introgression frequency >= 5%).

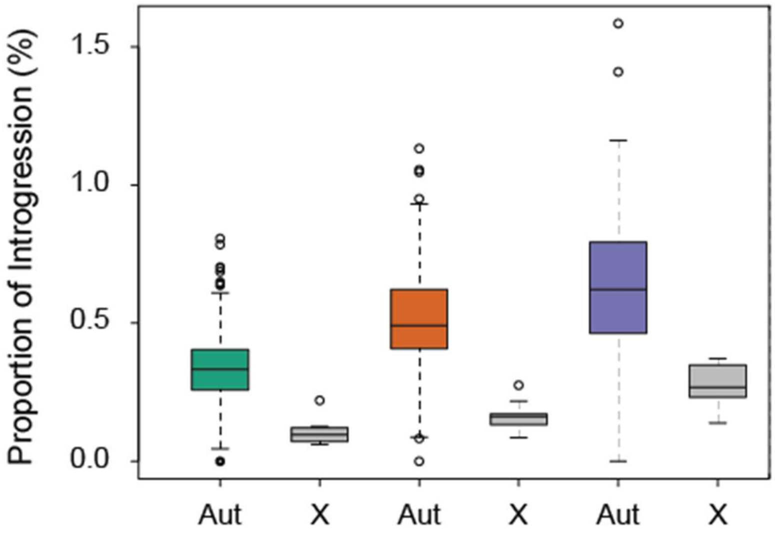

Figure S5. Variation of the proportion of introgression across individuals for autosomes (Aut) and X-chromosome (X) (Mann-Whitney U test p=0.00). From left to right, the results based on each of the three RND window sizes, 10kb (green), 20kb (orange) and 50kb (blue).

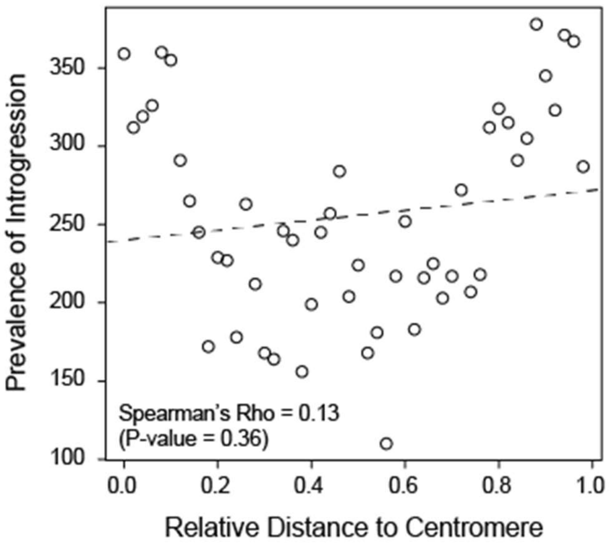

Figure S6. Correlation between prevalence of introgression (estimated with the ELAI method) and relative distance to the centromere (Spearman's rank correlation p=0.36). Dashed lines indicate a linear regression trendline. Only a subset of SNPs, at least 50kb apart from each other to avoid dependence, was considered.

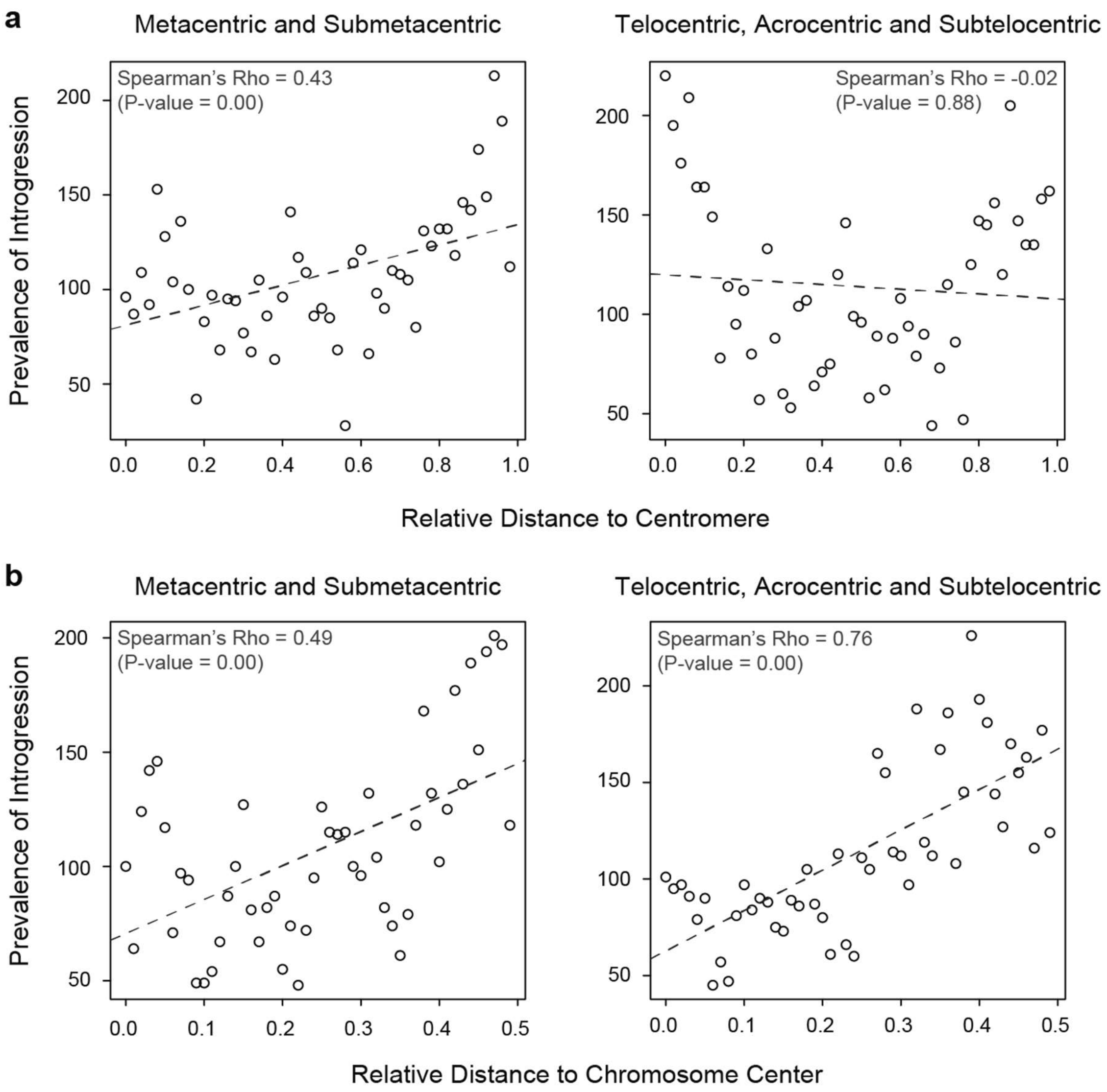

Figure S7. Correlation between prevalence of introgression (estimated with the ELAI method) and relative distance to (a) the centromere and (b) chromosome centre. Chromosomes were divided according to centromere position: metacentric/submetacentric (left panels) and telocentric/acrocentric/subtelocentric (right panels). Correlations were tested with Spearman's rank correlation test. Dashed lines indicate linear regression trendlines. Only a subset of SNPs, at least 50kb apart from each other to avoid dependence, was considered.

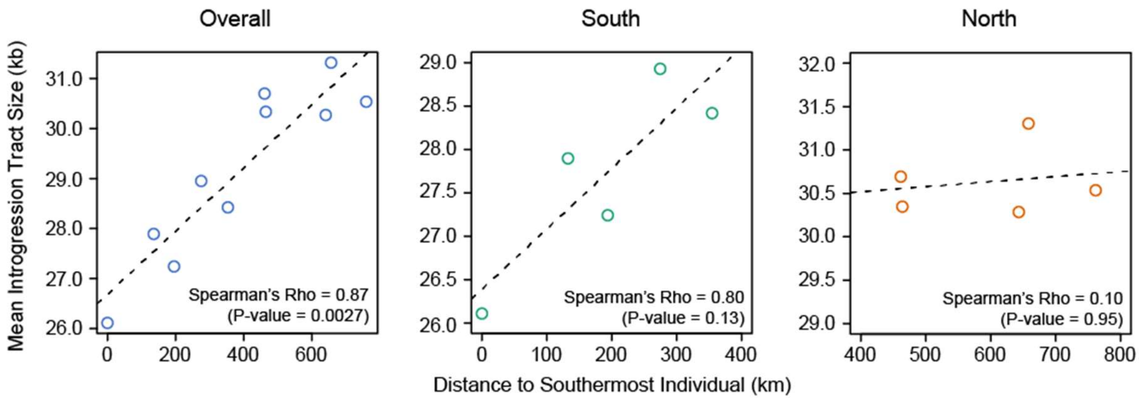

Figure S8. Correlation between introgression tract size and geography. For each of the 10 samples, mean introgression tract size (inferred with the ELAI method) is plotted against geographic distance to the southernmost sample (x axis). In the left panel all samples are considered, in the central panel only the 5 southern samples and in the right panel only the 5 northern samples. Correlations were tested with Spearman's rank correlation test. Dashed lines represent linear regression trendlines.

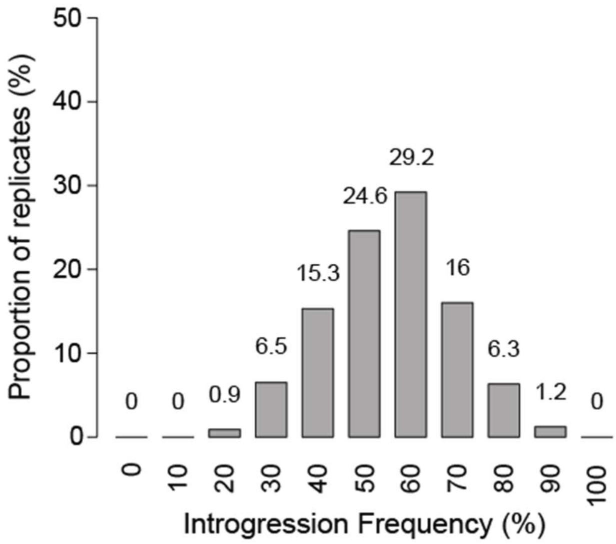

Figure S9. Expected introgression frequency distribution in a sample of 10 *L. granatensis* individuals with the same geographic origin as the 10 samples used in this study, supposing introgression frequencies of these population were as previously estimated for mtDNA in larger samples [45]. We simulated sampling of two haplotypes per population, with a probability of being introgressed equal to the empirical mtDNA introgression frequency of the population, and calculated introgression frequency over the 10 populations. The final distribution was built from a sample of 10,000 replicates.
